# How to Deal with Pulpitis: An Overview of New Approaches

**DOI:** 10.3390/dj13010025

**Published:** 2025-01-08

**Authors:** Jakub Fiegler-Rudol, Wojciech Niemczyk, Katarzyna Janik, Anna Zawilska, Małgorzata Kępa, Marta Tanasiewicz

**Affiliations:** 1 Department of Conservative Dentistry with Endodontics, Faculty of Medical Sciences in Zabrze, Medical University of Silesia, 41-902 Bytom, Polands80952@365.sum.edu.pl (K.J.); azawilska@sum.edu.pl (A.Z.); martatanasiewicz@sum.edu.pl (M.T.); 2 Department of Microbiology, Faculty of Pharmaceutical Sciences in Sosnowiec, Medical University of Silesia, 41-200 Sosnowiec, Poland; mkepa@sum.edu.pl

**Keywords:** regenerative endodontics, dental pulp, platelet-rich fibrin, tissue scaffolds, regeneration

## Abstract

**Background:** Traditional root canal therapy (RCT) effectively removes diseased or necrotic pulp tissue and replaces it with inorganic materials. Regenerative endodontics is an alternative to conventional RCT by using biologically based approaches to restore the pulp–dentin complex. This review explores emerging techniques, including autogenic and allogenic pulp transplantation, platelet-rich fibrin, human amniotic membrane scaffolds, specialized pro-resolving mediators, nanofibrous and bioceramic scaffolds, injectable hydrogels, dentin matrix proteins, and cell-homing strategies. These methods utilize stem cells, growth factors, and biomaterials to regenerate vascularized, functional pulp tissue. **Methods:** A narrative review was conducted using PubMed, Scopus, and Embase to identify studies published between 2010 and 2023. In vitro, animal, and clinical studies focusing on innovative regenerative endodontic techniques were analyzed. **Conclusions:** Although regenerative endodontics demonstrates great potential, challenges remain in standardizing protocols, addressing biological variability, and achieving consistent clinical outcomes. Future research must focus on refining these techniques to ensure their safety, efficacy, and accessibility in routine practice. By addressing current limitations, regenerative endodontics could redefine the management of pulpitis, offering biologically based treatments that enhance tooth vitality, structural integrity, and long-term prognosis.

## 1. Introduction

### 1.1. Rationale

The management of pulpitis has traditionally relied on root canal therapy (RCT), a well-established method for treating irreversible pulp inflammation and necrosis. However, RCT often involves replacing the diseased or necrotic pulp with inert materials, leading to the permanent loss of pulp vitality [1]. While effective in resolving infection and preserving the tooth, this approach can compromise long-term outcomes by reducing the tooth’s biomechanical strength and increasing the risk of fracture [2]. These limitations highlight the need for alternative therapies that not only address the underlying pathology but also aim to restore the functional integrity of the pulp–dentin complex. Recent advancements in regenerative endodontics have introduced biologically based approaches aimed at preserving or restoring pulp vitality in cases of irreversible pulpitis or necrosis [3]. Regenerative techniques seek to harness the body’s intrinsic healing potential, utilizing stem cells, growth factors, and biomaterials to promote tissue regeneration within the pulp space [3,4]. These methods offer the potential to improve treatment outcomes by preserving or restoring vascularization, neural function, and structural integrity, thus maintaining the tooth’s long-term functionality [2,3,5].

### 1.2. Objectives

The primary objective of this review is to provide an overview of new regenerative approaches in managing pulpitis, with a focus on techniques aimed at restoring the vitality and functionality of the pulp–dentin complex. Additionally, the review identifies existing knowledge gaps and outlines potential directions for future research to refine these techniques and ensure their accessibility in routine dental practice, ultimately redefining the standard of care in pulpitis treatment.

## 2. Materials and Methods

This narrative review aimed to summarize and analyze recent advancements in the treatment of pulpitis, with particular emphasis on regenerative approaches. Unlike systematic reviews with strict eligibility criteria, this broader narrative review sought to explore a wide range of regenerative endodontic techniques, evaluate their efficacy, and identify existing knowledge gaps. A comprehensive literature search was conducted in PubMed, Embase, and Scopus using both MeSH terms and free-text keywords (see Table 1 for search terms). The inclusion and exclusion criteria are detailed in Table 2. Publications in English from the last 15 years were included. Two authors (W.N. and J.F.-R.) independently screened the titles of retrieved records for relevance. From the selected articles, key data such as study design, sample size, regenerative methods, clinical outcomes, and techniques employed—ranging from stem cell transplantation and scaffolds to growth factors—were extracted. Outcomes pertaining to pulp vitality restoration, as well as any reported adverse effects and technique-specific limitations, were also documented.

## 3. Autogenic Dental Pulp Transplantation

Autogenic dental pulp transplantation involves transplanting healthy pulp tissue from a patient’s own tooth into another tooth, eliminating the risk of immune rejection and cross-infection [6]. This technique uses donor teeth extracted for non-pathological reasons, maintaining the pulp’s regenerative potential for revascularization and dentinogenesis. However, its limitations include limited donor availability and the risk of donor site morbidity such as discomfort or tooth loss [6,7]. Feitosa et al. demonstrated successful transplantation of a third molar pulp into premolars, observing maintained pulp vitality, reduced periapical lesions, and revascularization within 12 months [7]. Similarly, Haung et al., in a canine model, showed that autologous deciduous pulp transplantation in necrotic immature teeth reduced apical diameter and promoted dentin-like tissue formation compared to standard treatment [8]. Cehreli et al. applied regenerative endodontic treatment (RET) in traumatized incisors of children aged 8–11.5 years, using deciduous pulp as a scaffold [9]. Follow-ups revealed periapical healing, thickened dentinal walls, and progressive apical closure, highlighting autogenic pulp transplantation’s potential to restore pulp vitality and support root development in immature teeth [9].

## 4. Allogenic Dental Pulp Transplantation

Allogenic dental pulp transplantation involves transferring pulp tissue between two individuals of the same species [10]. Often, a donor tooth—such as a child’s deciduous tooth scheduled for extraction or a third molar—is used as the pulp source due to its rich content of regenerative stem cells and growth factors [11]. The recipient tooth, typically a damaged single-rooted permanent tooth in need of regenerative treatment, is prepared by thoroughly disinfecting and shaping the root canal [12]. The harvested donor pulp is then carefully placed into this prepared canal, followed by the application of biocompatible material to cover the transplanted pulp tissue and restorative procedures, often using resin-based materials [10,11]. This process aims to restore pulp vitality and encourage the formation of new, functional pulp tissue [10,11,12]. While allogenic pulp transplantation offers the advantage of utilizing readily available donor tissue rich in regenerative properties, its main drawbacks include the risk of immune rejection, limited donor availability, and potential cross-contamination, which require stringent clinical protocols to mitigate [10,11,12]. Feitosa et al. investigated allogenic pulp transplantation in three patient cases involving pulp from a child’s extracted teeth placed into a parent’s root canal. Two years of follow-up with imaging, pulp vitality tests, and Doppler ultrasound showed that all treated teeth eventually revascularized and exhibited no endodontic or periodontal radiolucency [10]. Although only a small number of cases were examined, the findings suggest that this technique could be a viable option for pulp revitalization. Notably, the protocol did not include apical bleeding or preventive antibiotic coverage [10].

## 5. Amniotic Membrane

Human amniotic membrane (HAM), a decellularized 3D scaffold derived from the placental layer, shows promise in regenerative endodontics due to its high biocompatibility, ability to promote cell adhesion, proliferation, migration, and capacity to act as a natural scaffold for pulp regeneration [13]. In vitro studies with human dental pulp stem cells (hDPSCs) demonstrate their role in enhancing tissue regeneration and vascularization, while in vivo studies report mild to moderate inflammation [14]. Both cell-free and cell-loaded HAM scaffolds support pulp-like tissue formation with revascularization and collagen deposition [4]. HAM offers advantages such as low immunogenicity, reducing rejection risks, and is readily available as a byproduct of childbirth. However, drawbacks include its mild inflammatory response, variability in bioactivity depending on source and preparation, and potential cost implications compared to traditional materials. Standardization and further long-term studies are needed to confirm its efficacy and cost-effectiveness [4,13,14,15]. Johri et al. demonstrated successful pulp regeneration using HAM after pulpotomy, showing normal pulp vitality and radiographic stability at an 18-month follow-up [13]. Saaid et al. found cryopreserved CAM to enhance odontogenic differentiation and cell attachment, with glycerol leading to higher ALP expression, suggesting strong regenerative potential [14]. Similarly, Joseph et al. reported root growth and apical closure in a traumatized immature incisor treated with HAM, reinforcing its clinical potential in regenerative endodontics [16].

## 6. Platelet-Rich Fibrin (PRF) and Platelet-Rich Plasma (PRP)

PRF and PRP are valuable tools in regenerative endodontics due to their ability to promote angiogenesis, collagen synthesis, and cell proliferation through growth factors like PDGF, TGF-β, and VEGF [17,18,19]. Niemczyk et al. demonstrated their antimicrobial effects against Porphyromonasgingivalis and their role in reducing bacterial load while stimulating tissue repair [17]. Additionally, injectable PRF (i-PRF) enhances dental pulp stem cell (DPSC) migration and differentiation, facilitating pulp regeneration and apical closure in immature necrotic teeth [18]. PRP provides anti-inflammatory properties, improving healing outcomes, while PRF ensures a sustained release of bioactive molecules for long-term tissue repair [17,18,19]. PRP and PRF have goof biocompatibility, autologous origin (eliminating immune rejection), and cost-effective, minimally invasive preparation [19]. PRP rapidly releases growth factors, accelerating healing, while PRF provides a slower, prolonged release [17,18,19]. However, the lack of standardized protocols for preparation and administration causes variability in outcomes, influenced by differences in centrifugation techniques and platelet concentration [17,18,19]. PRP’s effects may diminish over time, whereas PRF’s slower release may not suit immediate clinical applications [3]. Hosseini et al. reported successful regenerative endodontic treatment with advanced PRF+ (A-PRF+) in a 12-year-old patient with necrotic pulp and asymptomatic apical periodontitis. Over 24 months, the procedure led to complete symptom resolution, root development, and periapical healing, underscoring A-PRF+’s potential to restore tooth vitality in immature teeth [20].

## 7. Cell Homing Strategy and Stem Cells

Cell homing harnesses the body’s natural regenerative capabilities by attracting endogenous stem cells, such as DPSCs and SCAP, to damaged pulp tissue through chemotaxis [21,22]. Bioactive molecules, growth factors, and signaling proteins introduced into the root canal guide this process, while a blood clot or biocompatible materials provide a natural scaffold for cell migration and tissue regeneration [21,22,23,24]. The advantages of cell homing include its minimally invasive nature, avoidance of cell harvesting, and reduced risk of immune rejection [24]. Additionally, natural scaffolds like blood clots simplify clinical application [25]. However, outcomes depend heavily on the patient’s age, health, and microenvironmental factors [24]. Challenges include the need for precise delivery of growth factors, protocol standardization, and further clinical trials to ensure efficacy and reproducibility [25]. Nakashima et al. demonstrated the potential of mobilized dental pulp stem cells (MDPSCs) for pulp regeneration. In a pilot study, five patients with irreversible pulpitis underwent MDPSC transplantation with G-CSF in atelocollagen after pulpectomy. Within four weeks, pulp vitality was confirmed, and by 24 weeks, MRI and CBCT revealed regenerated pulp-like tissue and functional dentin formation, highlighting the safety and effectiveness of MDPSC-based therapies [26].

## 8. Nanofibrous Scaffolds

Nanofibrous scaffolds, composed of nanoscale fibers that mimic the extracellular matrix, are essential in regenerative endodontics [27]. They promote cell adhesion, proliferation, and differentiation while allowing nutrient and waste diffusion [27,28]. Fabricated from biocompatible materials like polycaprolactone or collagen, these porous, biodegradable scaffolds can incorporate growth factors for controlled release, supporting angiogenesis, odontogenesis, and tissue regeneration [29]. They can also serve as drug delivery systems for antimicrobial agents, enhancing clinical utility [27,28]. Advantages include their ability to mimic natural tissue structure, support cell growth, enable controlled growth factor release, and gradually degrade as new tissue forms, reducing the need for intervention [29]. Electrospinning allows for precise and customizable scaffold fabrication [27,28,29]. Disadvantages involve limited mechanical strength, complexity, and cost of fabrication [30,31]. Variability in growth factor release and drug delivery efficacy requires further optimization. Long-term clinical studies are also needed to confirm their safety and efficacy in endodontic practice [28,29]. Palasuk et al. developed PDS-based nanofibrous scaffolds with metronidazole and ciprofloxacin, demonstrating improved tensile strength, antimicrobial effects, and low cytotoxicity, highlighting their drug delivery potential [31]. Lovelace et al. showed that introducing blood clots, growth factors, and stem cells into immature teeth increased stem cell marker expression (CD73, CD105), enhancing pulp regeneration through evoked bleeding [32].

## 9. Bioceramic-Based Scaffolds

Bioceramics are biocompatible, inorganic materials widely used in regenerative endodontics, classified as bioinert, bioactive, or biodegradable [33]. Bioinert materials, like alumina and zirconia, remain biologically inactive, while bioactive materials, such as hydroxyapatite (HA) and calcium silicates, interact with tissues to promote healing and regeneration [34]. Biodegradable options, like tricalcium phosphate (TCP), integrate into tissue, enhancing natural regeneration [33,34,35]. Key advantages include excellent biocompatibility, osteoconductivity, and odontogenic differentiation potential, aided by the controlled release of ions like Si^4+^, PO_4_^3−^, and Ca^2+^, which stimulate biomineralization and tissue regeneration [35,36]. Bioceramics also form a hermetic seal with tooth tissues, making them ideal for pulp capping and root repair, while their radiopacity ensures accurate radiographic follow-ups [37]. However, limitations include their low resorption rate, especially with HA, which may lead to disorganized tissue and incomplete integration. Some bioceramics lack sufficient mechanical strength for load-bearing applications, and their fabrication costs can be high [37,38]. Further optimization of degradation profiles is necessary to achieve consistent clinical outcomes [39,40,41,42,43]. Calcium phosphate compounds (CPCs) like HA and TCP are commonly used in forms such as powder, granules, and blocks, proving effective for pulp-capping procedures by promoting the formation of dentin-like tissue [3]. While bioceramics show significant promise, ongoing research is needed to address these challenges and improve their clinical reliability [37,38,39,40,41,42,43].

## 10. Injectable Scaffolds and Stem Cells

Biodegradable hydrogels are a novel category of medical materials [44]. They consist of homopolymers, such as polylactic acid (PLA), polyglycolic acid (PGA), and polycaprolactone (PCL), and co-polymers, such as poly(lactide-co-glycolide)-polyethene glycol (PEG-PLGA) and L- and DL-lactide (PLDLA) [44,45]. Hydrogels are injectable scaffolds that can be delivered by syringe, making them potentially noninvasive and easy to administer into root canal systems [45]. The hydrogel can facilitate pulp regeneration by providing a substrate for cell proliferation and differentiation into an organized tissue structure [4]. The lesions can biodegrade within a short period of weeks or months, converting to carbon dioxide and water [44]. This process allows natural tissue to fill the space previously occupied by the lesions. However, these polymers lose strength before they lose mass [44,45,46].

Early ingrowth of natural tissue is inhibited, and subsequent rapid mass loss can cause inflammation due to the production of acidic degradation products [44,45]. Puramatrix™ is a liquid that can be poured into a pulp chamber and self-polymerizes under physiological conditions to form a solid gel that can support cell growth [45]. This application is appealing from an endodontic perspective because a liquid can conform more easily to the variable shape of a pulp chamber than a solid or moldable scaffold [44]. According to Cavalcanti et al. (2013), dental pulp stem cells can survive and proliferate in a 3D Puramatrix™ scaffold [46].

## 11. Dentin Matrix Proteins

Dentin matrix proteins (DMPs) are key bioactive components in regenerative endodontics, promoting the formation and regeneration of dental tissues, particularly dentin [47]. Derived from dentin’s extracellular matrix, DMPs include collagen and non-collagenous proteins like dentin sialoprotein (DSP), dentin phosphoprotein, and MEPE, which provide mechanical support and biochemical signals to guide mineralization and odontogenic differentiation [48]. They also regulate cell adhesion, proliferation, and differentiation, ensuring reparative dentin formation [47,48]. The advantages of DMPs include their ability to stimulate dentin-like matrix regeneration and maintain pulp vitality, making them valuable for direct pulp capping and vital pulp therapy. Combined with small extracellular vesicles (sEVs) from dental pulp cells, DMPs enhance cell proliferation, migration, and reparative dentin growth, as shown in vivo with TDM-sEV complexes. Bone morphogenetic proteins (BMPs), especially BMP-2, further boost odontoblastic differentiation of stem cells from exfoliated deciduous teeth (SHED) [49]. However, DMPs face challenges, including costly isolation and purification, variability in biological activity, and the need for precise BMP-2 regulation to avoid ectopic mineralization [47,48,49]. Long-term clinical efficacy and safety remain to be validated [49,50]. Integrating DMPs, BMPs, and bioactive components into engineered scaffolds and stem cell therapies can establish a regenerative microenvironment, advancing dentin–pulp complex regeneration. Future research should address current limitations to optimize their clinical application [50,51].

## 12. Resolvin E1

Endogenous specialized pro-resolving mediators (SPMs), such as resolvins, lipoxins, protectins, and maresins, play a key role in inflammation resolution and tissue repair [51]. Resolvin E1 (RvE1), a major omega-3 fatty acid metabolite, has shown promise in managing pulp inflammation and promoting reparative dentin formation by inhibiting NF-κB activation, reducing pro-inflammatory factors, and preventing ectopic mineralization [51,52,53,54,55]. RvE1 enhances odontoblastic differentiation, proliferation, and chemotaxis of dental pulp stem cells (DPSCs), ensuring targeted resolution of inflammation and pulp regeneration [52]. Advantages of this approach include its ability to resolve inflammation without suppressing healing responses, promote dentin formation, and prevent ectopic mineralization in a minimally invasive manner [53,54,55]. However, limitations include the need for further clinical validation, challenges in delivering consistent concentrations, its short half-life, and the high cost of production, which may impact accessibility [51,52,53,54,55]. While promising, further research is essential to optimize its clinical application and long-term efficacy [55].

## 13. Conclusions

Regenerative endodontics represents a shift in the treatment of pulpitis by focusing on restoring the pulp–dentin complex through biologically driven approaches rather than conventional root canal therapy. This review highlights promising techniques such as autogenic and allogenic pulp transplantation, PRF, human amniotic membrane scaffolds, cell-homing strategies, and nanofibrous and bioceramic scaffolds, all of which leverage stem cells, growth factors, and biocompatible materials to promote tissue regeneration, vascularization, and functional recovery. A detailed summary of these methods is provided in Table 3. These advancements offer significant potential for preserving tooth vitality and structural integrity while reducing long-term complications. However, key challenges remain, including the need for standardized protocols, consistent clinical outcomes, and further validation through robust, long-term clinical studies to address biological variability and ensure safety, efficacy, and cost-effectiveness. By overcoming these limitations and refining these techniques, regenerative endodontics has the potential to establish itself as the gold standard for managing pulpitis, ultimately improving patient outcomes and redefining the future of endodontic care.

## Figures and Tables

**Table 1:** Search syntax used in the study.

Source	Search Term
PubMed/MEDLINE	(“Pulpitis”[Mesh] OR pulpitis[tiab] OR “diseased pulp”[tiab] OR “pulp inflammation”[tiab]) AND (“Regenerative Endodontics”[Mesh] OR “pulp regeneration”[tiab] OR “stem cell therapy”[tiab] OR “stem cells”[Mesh] OR “platelet-rich fibrin”[tiab] OR “amniotic membrane”[tiab] OR “specialized pro-resolving mediators”[tiab] OR “bioceramic scaffold”[tiab] OR “nanofibrous scaffold”[tiab] OR “dentin matrix proteins”[tiab] OR “dental pulp stem cells”[tiab]) AND (“Dental Pulp”[Mesh] OR “dental pulp”[tiab] OR “pulp tissue”[tiab])
Embase	(‘pulpitis’/exp OR pulpitis:ti,ab OR ‘diseased pulp’:ti,ab OR ‘pulp inflammation’:ti,ab) AND (‘regenerative endodontics’/exp OR ‘pulp regeneration’:ti,ab OR ‘stem cell therapy’:ti,ab OR ‘stem cells’/exp OR ‘platelet-rich fibrin’:ti,ab OR ‘amniotic membrane’:ti,ab OR ‘specialized pro-resolving mediators’:ti,ab OR ‘bioceramic scaffold’:ti,ab OR ‘nanofibrous scaffold’:ti,ab OR ‘dentin matrix proteins’:ti,ab OR ‘dental pulp stem cells’:ti,ab) AND (‘dental pulp’/exp OR ‘dental pulp’:ti,ab OR ‘pulp tissue’:ti,ab)
Scopus	(TITLE-ABS-KEY (pulpitis) OR TITLE-ABS-KEY (“diseased pulp”) OR TITLE-ABS-KEY (“pulp inflammation”)) AND (TITLE-ABS-KEY (“regenerative endodontics”) OR TITLE-ABS-KEY (“pulp regeneration”) OR TITLE-ABS-KEY (“stem cell therapy”) OR TITLE-ABS-KEY (“stem cells”) OR TITLE-ABS-KEY (“platelet-rich fibrin”) OR TITLE-ABS-KEY (“amniotic membrane”) OR TITLE-ABS-KEY (“specialized pro-resolving mediators”) OR TITLE-ABS-KEY (“bioceramic scaffold”) OR TITLE-ABS-KEY (“nanofibrous scaffold”) OR TITLE-ABS-KEY (“dentin matrix proteins”) OR TITLE-ABS-KEY (“dental pulp stem cells”)) AND (TITLE-ABS-KEY (“dental pulp”) OR TITLE-ABS-KEY (“pulp tissue”))

**Table 2:** Selection criteria for papers included in this review.

Inclusion Criteria	Exclusion Criteria
- Preclinical studies - Clinical studies - Review articles and meta-analyses relevant to pulp regeneration - Recent and relevant studies about regeneration techniques for both pulpitis and necrotic teeth - Investigations of regenerative endodontic strategies - Outcomes related to pulp vitality restoration, healing success, cellular and molecular mechanisms, and tooth structural integrity improvements - Articles published in English within the last 15 years	- Studies not related to regenerative endodontics or pulpitis - Editorials, commentaries, opinion pieces, and gray literature - Studies without specific regenerative outcomes or biological mechanisms - Articles with incomplete or inaccessible data - Publications not available in full text - Articles in languages other than English without translation - Articles older than 15 years

**Table 3:** Summary of the discussed methods.

Method	Description	Advantages	Disadvantages	Clinical Outcomes
Autogenic Dental Pulp Transplantation [6,7,8,9]	Transplanting healthy pulp tissue from a patient’s own tooth into another.	No immune rejection, promotes revascularization and dentinogenesis.	Limited donor availability, risk of donor site morbidity.	Maintains pulp vitality, promotes dentin-like tissue formation.
Allogenic Dental Pulp Transplantation [10,11,12]	Transferring pulp tissue between individuals of the same species.	Readily available donor pulp, rich in stem cells and growth factors.	Risk of immune rejection, stringent protocols needed.	Restored pulp vitality, revascularization, and healing observed.
Amniotic Membrane [13,14,16]	Using a decellularized human placental membrane as a scaffold.	High biocompatibility, promotes cell adhesion and vascularization.	Mild inflammation, variable bioactivity, potential high cost.	Supports pulp regeneration, root growth, and apical closure.
Platelet-Rich Fibrin (PRF) and Platelet-Rich Plasma (PRP) [17,18,19,20]	Using autologous platelet derivatives to promote regeneration and healing.	Autologous origin, promotes angiogenesis and tissue repair.	Lack of standard protocols, variability in outcomes.	Promotes pulp regeneration, root development, and healing.
Cell Homing Strategy and Stem Cells [21,22,23,24,25,26]	Attracting endogenous stem cells to regenerate pulp tissue naturally.	Minimally invasive, avoids cell harvesting, uses natural scaffolds.	Depends on patient’s health, requires precise growth factor delivery.	Confirmed pulp vitality, pulp-like tissue regeneration.
Nanofibrous Scaffolds [27,28,29,30,31,32]	Scaffolds made of nanoscale fibers mimicking the extracellular matrix.	Mimics natural tissue, supports cell growth, controlled degradation.	Limited mechanical strength, costly fabrication, variability in release.	Enhanced pulp regeneration, antimicrobial properties.
Bioceramic-Based Scaffolds [33,34,35,36,37,38,39,40,41,42,43]	Biocompatible materials (e.g., calcium silicates) promoting tissue regeneration.	Excellent biocompatibility, promotes mineralization and healing.	Low resorption rate, limited mechanical strength, high cost.	Effective pulp capping, dentin-like tissue formation.
Injectable Scaffolds and Stem Cells [43,44,45,46]	Injectable hydrogels for delivering cells and growth factors.	Non-invasive, conforms to pulp chamber shape, promotes regeneration.	Rapid degradation may cause inflammation, requires optimization.	Supports cell proliferation, differentiation, and tissue repair.
Dentin Matrix Proteins [46,47,48,49,50,51]	Bioactive components derived from dentin’s extracellular matrix.	Stimulates dentin-like matrix regeneration, enhances cell growth.	Costly isolation, variability in activity, risk of ectopic mineralization.	Promotes reparative dentin formation, maintains pulp vitality.
Resolvin E1 [52,53,54,55]	Omega-3-derived lipid mediator for inflammation resolution and pulp repair.	Resolves inflammation, promotes dentin formation, minimally invasive.	Short half-life, high cost, requires consistent delivery.	Inhibits inflammation, promotes pulp regeneration.

## Data Availability

This review analyzed already existing data, obtained from the cited literature.
